# Multiscale Cooperative Differential Evolution Algorithm

**DOI:** 10.1155/2019/5259129

**Published:** 2019-12-17

**Authors:** Yongzhao Du, Yuling Fan, Xiaofang Liu, Yanmin Luo, Jianeng Tang, Peizhong Liu

**Affiliations:** ^1^College of Engineering, Huaqiao University, Quanzhou 362021, China; ^3^College of Computer Science and Technology, Huaqiao University, Xiamen 361021, China; ^2^Research Center of Apply Statistics and Big Data, Huaqiao University, Xiamen 361021, China

## Abstract

A multiscale cooperative differential evolution algorithm is proposed to solve the problems of narrow search range at the early stage and slow convergence at the later stage in the performance of the traditional differential evolution algorithms. Firstly, the population structure of multipopulation mechanism is adopted so that each subpopulation is combined with a corresponding mutation strategy to ensure the individual diversity during evolution. Then, the covariance learning among populations is developed to establish a suitable rotating coordinate system for cross operation. Meanwhile, an adaptive parameter adjustment strategy is introduced to balance the population survey and convergence. Finally, the proposed algorithm is tested on the CEC 2005 benchmark function and compared with other state-of-the-art evolutionary algorithms. The experiment results showed that the proposed algorithm has better performance in solving global optimization problems than other compared algorithms.

## 1. Introduction

The differential evolution (DE) is a bionic intelligence method proposed by American scholars Rainer Storn and Kenneth Price in 1995, simulating survival of the fittest [1, 2]. The algorithm adopts mutation, crossover, and selection operations to mimic genetic mutations during biological evolution and retains highly adaptable individuals for optimal solutions. Aiming at the problems of both population convergence stagnation and premature convergence, researchers mainly focus on three aspects of control parameter setting and mutation strategy selection [3–6], crossover operation [7–9], and population structure [10–12] to improve the algorithm performance. The DE has been widely concerned by researchers because of its simple coding, convergence, and strong robustness. It has been applied in many fields such as industrial control [13], antenna design [14], power system [15], image processing [16], and so on.

The parameter control and evolutionary strategy selection are mainly discussed in two aspects of DE. On the one hand, control parameter settings for the scaling factor *F*, crossover probability CR, and population size NP [17]. On the other hand, different strategies for different optimization problems [18], we need to choose the most suitable strategy. The parameter setting affects the population diversity [19], the development ability of the early period, and the convergence of the later period [20]. The choice of evolutionary strategy is the key step to determine the balance between exploration and convergence of DE, and different evolution strategies will show different surveying capabilities and the convergence tendencies. At the same time, diverse crossover operations have diverse effects on seeking global optimization. Although the traditional binomial crossover operation has a certain role, it is more dependent on the cross coordinate system and is widely used. In addition, the population structure is also an important indicator for the algorithm performance. If the population size is too small, it will easily lead to the loss of effective alleles, thereby reducing the generation of competitive individuals. In contrast, if the population size is too large, the possibility of correct search direction by the algorithm will be reduced [11].

Due to premature convergence, parameter control, and strategy improvement, crossover operation and population structure attract increasing attention to improve the performances of DEs [17, 21–24]. Therefore, numerous DE-improvement algorithms [25, 26] have been proposed constantly based on parameters and strategies, such as parameter-adaptive jDE [21], JADE using “current-to-pbest/1” strategy and adaptive parameters [24], SaDE using adaptive difference strategy [23], CoDE suiting for experimental individual algebra strategy and control parameters [27], EPSDE for mutation strategy and control parameter [17], TDE for triangular mutation strategy [28], and super-fit multistandard adaptive SMADE [29]. Moreover, the DE is improved based on the crossover operation, such as ODE [30] adopting orthogonal crossover operators and CoBiDE [31] using covariance learning and bimodal distribution of parameters. And DE is improved based on population structure, such as SPSRDEMMS [32] for multivariation strategies, MPEDE [33] for multipopulations and strategy sets, master-slave model [34], island model [35], cellular model [36], level model [37], and pool model [38]. In recent years, population segregation techniques have been used to improve evolutionary algorithms, including particle swarm optimization, genetic algorithm, and evolutionary algorithm [39–44].

To further improve the convergence and reduce the population stagnation, a multiscale cooperative differential evolution (MCDE) algorithm is proposed. In terms of parameter setting, the scaling factor *F* and the crossover probability CR are mainly adjusted based on the literature [24]. In the selection of mutation strategy, the MCDE selects “current-to-pbest/1,” “current-to-rand/1,” and “rand/1” as mutation strategy groups. In the initial phase, the evolutionary population was divided into multiple subpopulations, and one subpopulation was selected as the experimental population to test the mutation strategy with better evolutionary results. In the evolutionary phase, the global search capability is continuously promoted by establishing a constant rotation of the cross coordinate system and coordinating among multiple subpopulations. In the end, the best individual that remains is used as the optimal solution. In CEC 2005, 30-dimension and 50-dimension simulation tests were conducted and compared with contemporary evolutionary algorithms; MCDE was found to have more significant effects.

The paper is organized as follows.  Section 2 briefly introduces the standard DE algorithm.  Section 3 elaborates on the algorithm improvement.  Section 4 analyzes the significance of the proposed algorithm through experimental data.  Section 5 gives a summary.

## 2. Standard Differential Evolution Algorithm

DE can be regarded as greedy evolution algorithm based on real number coding and global optimization. In the evolutionary phase, three iteration processes of mutation, crossover, and selection are performed until the stop condition is satisfied. The fitness function *f*(*x*) is utilized to evaluate the quality and the best individual is recorded.

### 2.1. Initialization

Assuming that the population size is NP and the dimension of the feasible solution space is *D*, *x*^*G*^ is employed to represent the evolution population of *G* generation. Each individual is composed of *D*-dimensional parameters, which can be expressed as

(1) $$\begin{array}{c} x_{i}^{G}=\left\{x_{i,1}^{G},x_{i,2}^{G},\ldots ,x_{i,D}^{G}\right\},\;i\in \left\{1,2,\ldots ,\text{NP}\right\}, \end{array}$$

where *x*_*i*,*j*_^*G*^ ∈ (*x*^L^,  *x*^H^) and *x*^L^ and *x*^H^ represent the upper and lower bounds of the individual, respectively.

### 2.2. Mutation Operation

The individual *x*_*i*_^*G*^ in the parent population generates a variant individual *v*_*i*_^*G*^ by a mutation strategy. “DE/rand/1” indicates that the DE chooses a random perturbation individual to mutate. The expression is as follows:

(2) $$\begin{array}{c} v_{i}^{G}=x_{r1}^{G}+F\cdot \left(x_{r2}^{G}-x_{r3}^{G}\right),\;r1,r2,r3\in \left\{1,2,\ldots ,\text{NP}\right\}, \end{array}$$

where *r*1 ≠ *r*2 ≠ *r*3 and *r*1, *r*2, and *r*3 are a randomly generated mutation individual. The scaling factor *F* is chosen from [0, 1].

### 2.3. Crossover Operation

The main function of the crossover operation is that the generated variant individuals cross with individuals in the original population to generate new crossover individuals. The DE adopts binomial crossover scheme. The crossover operation is as follows:

(3) $$\begin{array}{c} u_{i,j}^{G}=\left\{\begin{array}{cc} v_{i,j}^{G}, & \text{if }{\text{rand}}_{j}\leq \text{CR or }j=j_{\text{rand}},\\ x_{i,j}^{G}, & \text{otherwise,}j=1,\ldots ,D, \end{array}\right. \end{array}$$

where rand_*j*_∈ [0, 1], *j*_rand_ is chosen from {1, 2,…, *D*}, and crossover probability *CR* is [0, 1].

### 2.4. Selection Operation

The selection operation mainly adopts the greedy selection mode of the survival of the fittest, making the offspring always superior to or equal to the parent individual *x*_*i*_. When the fitness value of the new individual *u*_*i*_ is better than that of the objective individual, the new individuals *u*_*i*_ will be accepted by the population. Otherwise, *x*_*i*_ still remains in the next generation population and continues to perform mutation and crossover operations as the objective individual in the next iterative calculation so that the population will always evolve toward the optimal solution. The selection operation is for minimization fitness value as follows:

(4) $$\begin{array}{c} x_{i}^{G+1}=\left\{\begin{array}{cc} u_{i}^{G}, & \text{if }f\left(u_{i}^{G}\right)\leq f\left(x_{i}^{G}\right),\\ x_{i}^{G},\; & \text{otherwise,} \end{array}\right. \end{array}$$

where *f*(*x*) is the objective function to be optimized.

## 3. Multiscale Cooperative Differential Evolution Algorithm

In the proposed algorithm, we divide the whole population into multiple subpopulations and give corresponding mutation strategies. Then, the cross coordinate system of each subpopulation is established by covariance learning and parameter adaptation of evolutionary subpopulation. Finally, the obtained crossover individual is selected and the individuals with better fitness are retained to make the whole population search forward to the global optimal solution.

### 3.1. Multiscale Mutation Strategy Integration Method

In recent years, because different mutation strategies are suitable for solving different optimization functions, some researchers mainly focus on multiple mutation strategies method [23, 24]. Even for a specific optimization problem, the most appropriate mutation strategy may be different at different stages of evolution. Therefore, mutation strategy is an important indicator to ensure significant results in the DE. During evolution, this paper selects “current-to-pbest/1,” “current-to-rand/1,” and “rand/1” as the multiscale mutation strategy set because of the different performance requirements for the mutation strategy. The individuals of “current-to-rand/1” and “rand/1” involved in mutation are all selected in a random manner so that global optimization can be performed in the early stages of evolution. “current-to-pbest/1” seeks the global optimal solution through the current best population individual. During evolution, the search range can be reduced to the vicinity of the optimal solution and the convergence speed can be accelerated.

Current-to-pbest/1:

(5) $$\begin{array}{c} v_{i}^{G}=x_{i}^{G}+F\cdot \left(x_{\text{pbest}}^{G}-x_{i}^{G}+x_{r1^{i}}^{G}-x_{r2^{i}}^{G}\right). \end{array}$$

Current-to-rand/1:

(6) $$\begin{array}{c} v_{i}^{G}=x_{i}^{G}+K\cdot \left(x_{r1^{i}}^{G}-x_{i}^{G}\right)+F\cdot \left(x_{r2^{i}}^{G}-x_{r3^{i}}^{G}\right). \end{array}$$

rand/1:

(7) $$\begin{array}{c} v_{i}^{G}=x_{r1^{i}}^{G}+F\cdot \left(x_{r2^{i}}^{G}-x_{r3^{i}}^{G}\right), \end{array}$$

where *x*_pbest_^*G*^ is uniformly chosen as one of the top *p* individuals in the current population with pbest ∈ (0, 1]. Because the three mutation strategies have their own advantages, there are some differences. Therefore, the population multiscale mechanism is introduced in this paper. The whole population Pop is divided into three subpopulation Pop_1_, Pop_2_, and Pop_3_. Pop_1_ with the largest population size is determined as the experimental population and combined with the corresponding mutation strategy. During evolution, the experimental population is allocated to mutation strategy with better evolution results. The population structure is expressed as follows:

(8) $$\begin{array}{c} \text{Pop}=\underset{i=1,2,3}{\sum }{\text{Pop}}_{i},\\ {\text{NP}}_{i}=\sigma_{i}*\text{NP,}\\ \underset{i=1,2,3}{\sum }\sigma_{i}=1, \end{array}$$

where we assume that Pop_1_ is an experimental population, NP is the population size, *σ* represents population size ratio, *σ*_1_ > *σ*_2_=*σ*_3_ and *σ*_*i*_∈ [0, 1].

After the population structure is well designed, the distribution rules of the subpopulations should be given. First, subpopulation Pop_1_, Pop_2_, and Pop_3_ incorporate corresponding mutation strategies. Then, the population undergoes mutation, crossover, and selection operations. Finally, the total number bd_*i*_ of superior individuals retained after each subpopulation evolution is counted. That is, the superior rate br_*i*_ of the subpopulation can be expressed as

(9) $$\begin{array}{c} {\text{br}}_{i}=\frac{{\text{bd}}_{i}}{{\text{NP}}_{i}},\;i=1,2,3. \end{array}$$

The superior rate br_*i*_ of each generation of subpopulation is calculated, and the subpopulation is reallocated for three mutation strategies according to the superior rate br_*i*_ in the next generation initialization stage. The multiscale mutation strategy set method makes full use of the advantages of the three mutation strategies to regulate and balance the contradiction between the population diversity and the convergence speed, which can be seen from the experimental results of the latter. In the first generation, the subpopulations randomly assign a mutation strategy. At the end of the first generation, we calculate the subpopulations superior rate by equation (9). The maximum superior rate stands for the best mutation strategy in this generation. Assume that the first-generation mutation strategy “current-to-pbest/1” has the highest superior rate, and the second generation assigns Pop_1_ to it. The remaining subpopulations Pop_2_ and Pop_3_ randomly assign a mutation strategy (“current-to-rand/1” or “rand/1”).

### 3.2. Covariance Learning

The aforementioned crossover operators mainly depend on the coordinate system, while the distribution information of the population reflects the direction of evolution to some extent [20]. During evolution, the distribution of population is often neglected, leading to the possibility of the population falling into local optimum and premature convergence. In this paper, variance and covariance are utilized to analyze population distribution and form covariance matrix to reflect population diversity information. Therefore, the systematic use of covariance matrix can reduce the dependence on coordinate system and the interaction between variables. Covariance matrix learning includes two related technologies: the feature decomposition and coordinate transformation of covariance matrix. The covariance matrix learning steps are as follows:

*Step 1*. Calculate covariance matrix C of subpopulations.
*Step 2*. Get the eigenvalue λ and feature vector matrix R of covariance.
*Step 3*. Update the objective individual and the variant individual through the feature-based cooperative system.
  (10) $$\begin{array}{c} \left\{\begin{array}{c} x_{i}^{G}=R^{-1}x_{i}^{G}=R^{T}x_{i}^{G},\\ v_{i}^{G}=R^{-1}v_{i}^{G}=R^{T}v_{i}^{G}. \end{array}\right. \end{array}$$
*Step 4*. Populations with better fitness for crossover and selection operations are retained and rotated back to the original coordinate system.
  (11) $$\begin{array}{c} u_{i}^{G}=R\cdot u_{i}^{G}. \end{array}$$

**Figure 1:**
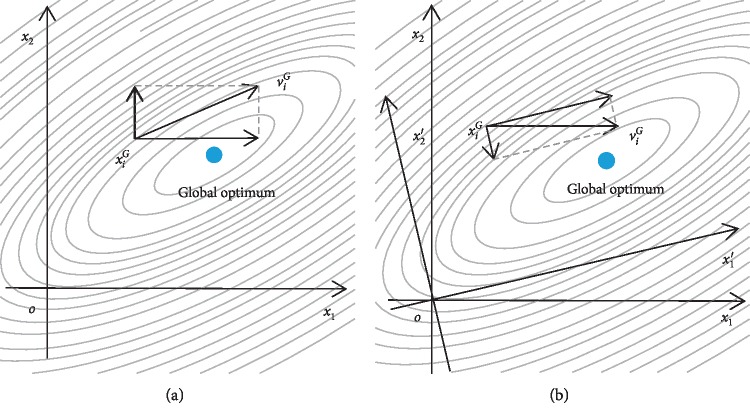
Population initial coordinate system and population feature coordinate system: (a) the initial coordinate system of population evolution; (b) the Eigen coordinate system of population evolution.

Based on the above four steps, we establish the population feature coordinate system.  Figure 1(a) shows the initial coordinate system of population evolution, and Figure 1(b) shows the feature coordinate system. By analyzing the population feature, we obtain the o*x*_1_*x*_2_ coordinate system and discover that we can find the global optimum faster.

### 3.3. Adaptive Control Parameter Settings

At present, researchers have proposed many effective parameter adaptation methods [21, 23, 24]. The combination of different control parameters and mutation strategies for the optimization problem will yield different results. In this paper, each scale strategy has its own control parameters, and different technologies are applied to the algorithm. The method in [24] is more suitable for the algorithm, so it adapts to the algorithm by improving its technology.

During evolution, scaling factor *F* plays a decisive role in the search range of base vectors. In standard DE algorithm, the value of *F* is a fixed value, which cannot be applied to solve all global optimization functions. In this paper, the scalar factor *F* mainly adopts the Cauchy inverse cumulative distribution function, assuming that *F*_*i*,*j*_ represents the scale factor of each dimension in the individual. *F*_*i*,*j*_ is expressed as follows:

(12) $$\begin{array}{c} F_{i,j}={\text{Cauchy}}_{i,j}\left(Fm_{j},0.1\right), \end{array}$$

where *Fm*_*j*_ is the position parameter of the Cauchy inverse cumulative distribution function and the scale factor of current individual and the initial value of *Fm*_*j*_ is set to 0.5. 0.1 indicates the scale parameter of the Cauchy inverse cumulative distribution function. To better apply to population evolution, the weighting factor *c* is introduced to combine the parent factor and the next generation factor. The current *Fm*_*j*_ is expressed as follows:

(13) $$\begin{array}{c} Fm_{j}=\left(1-c\right)\cdot Fm_{j}+c\cdot {\text{mean}}_{F}\left(S_{F,j}\right), \end{array}$$

where *c* ∈ [0,1] and parental scalar factor *S*_*F*,*j*_ is calculated using the power mean. The power mean is expressed as follows:

(14) $$\begin{array}{c} {\text{mean}}_{F}\left(S_{F,j}\right)={\left(\frac{1}{\text{NP}}\cdot \underset{\text{NP}}{\sum }F^{n}\right)}^{1/n}, \end{array}$$

where *n* is the index value of the power mean, which is quantified to the influence of the parent's scaling factor on the offspring.

In the DE algorithm, the crossover probability CR determines the possibility that an objective individual inherits gene from variant individual *v*_*i*_^*G*^. In this paper, the crossover probability CR mainly adopts the normal distribution function, assuming that CR_*i*,*j*_ represents the crossover probability of each dimension in the individual. CR_*i*,*j*_ is expressed as follows:

(15) $$\begin{array}{c} {\text{CR}}_{i,j}=\text{rand }n_{i,j}\left(\text{CR}m_{j},0.1\right), \end{array}$$

where CR*m*_*j*_ is the mean of individual crossover probability and the initial value is set to 0.5. The standard deviation of normal distribution is set to 0.1. To better inherit the parent gene, a weighting factor *c* is introduced to combine the parent crossover probability with the next generation crossover probability. CR*m*_*j*_ is expressed as follows:

(16) $$\begin{array}{c} \text{CR}m_{j}=\left(1-c\right)\cdot \text{CR}m_{j}+c\cdot {\text{mean}}_{\text{CR}}\left(S_{\text{CR},j}\right), \end{array}$$

where *c* ∈ [0,1] and parental crossover probability *S*_CR,*j*_ is calculated using the Lehmer mean. The Lehmer mean is as follows:

(17) $$\begin{array}{c} {\text{mean}}_{\text{CR}}\left(S_{\text{CR},j}\right)=\frac{\sum_{D}{\text{CR}}^{2}}{\sum_{D}\text{CR}}. \end{array}$$

The Lehmer mean method can flexibly adjust the value of CR according to the parent cross probability.

### 3.4. Algorithm Framework

The proposed algorithm combines multiscale strategy and covariance learning and introduces adaptive control parameters to lead the population to keep close to the global optimum. Based on the above analysis, the basic flow of MCDE is summarized as Algorithm 1.

## 4. Experimental Results and Analysis

The MCDE is tested on 25 benchmark functions of IEEE CEC 2005. The 25 benchmark functions mainly include unimodal function *F*_1_–*F*_5_, basic multimodal function *F*_6_–*F*_12_, extended multimodal function *F*_13_–*F*_14_, and complex function *F*_15_–*F*_25_. For details, please refer to [45]. In this paper, the parameter setting of the MCDE is as follows: population size NP = 250 and subpopulation ratio *σ*_1_ = 0.6, *σ*_2_ = *σ*_3_ = 0.2. Experimental environment: the operating system is win7 Professional 64 bit, CPU is core i7 (3.40 GHz), RAM is 8 GB, and the compiler is MATLAB R2014b.

### 4.1. Comparison with Improved DE Algorithm

**Figure 2:**
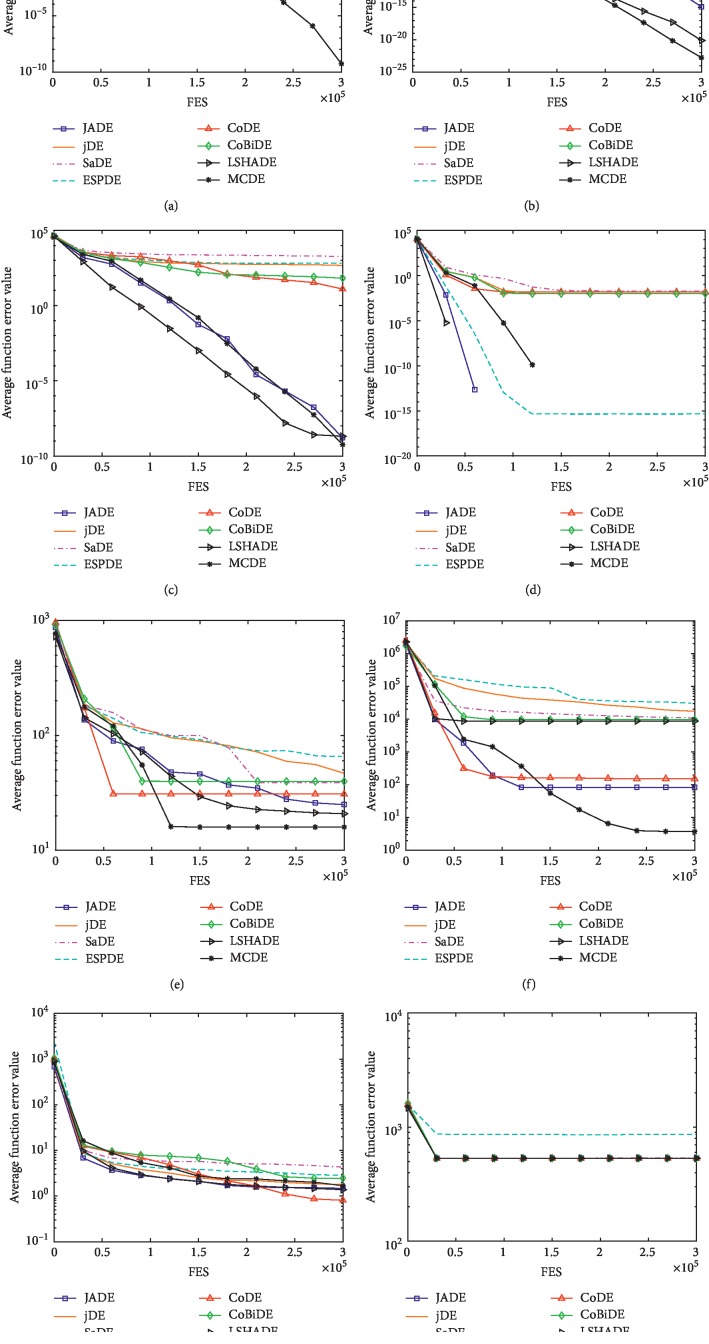
Evolution of the mean function error values derived from JADE, jDE, SaDE, CoDE, CoBiDE, LSHADE, and MCDE versus the number of FES on *F*_3_, *F*_4_, *F*_5_, *F*_7_, *F*_10_, *F*_12_, *F*_13_, and *F*_23_. (a) *D* = 30 *F*_3_. (b) *D* = 30 *F*_4_. (c) *D* = 30 *F*_5_. (d) *D* = 30 *F*_7_. (e) *D* = 30 *F*_10_. (f) *D* = 30 *F*_12_. (g) *D* = 30 *F*_13_. (h) *D* = 30 *F*_23_.

To verify the performance of the MCDE, it is compared with the six classic DE-improvement algorithms: JADE [24], jDE [21], SaDE [23], EPSDE [17], CoDE [18], CoBiDE [31], and LSHADE [46]. JADE and jDE are representative algorithms and are heavily referenced. SaDE and EPSDE are based on multistrategy improved algorithms. CoDE and CoBiDE are improved algorithms based on population structure. The experimental results of the above seven algorithms are shown in Table 1, where *D* = 30 and MaxFES = 300000. The form of the numerical values in the table is the mean error ± standard deviation. “−/+/≈” means that the comparison algorithm is obviously better than, worse than, and similar to MCDE. Based on the data given in Table 1 and Figure 2, we can draw the following conclusions: (1) unimodal function *F*_1_–*F*_5_: among the comparison algorithms, JADE and LSHADE have the best effect. Because of the greedy strategy “current-to-pbest/1,” the algorithm can achieve fast convergence and high precision. However, the MCDE multiscale strategy achieves better results than JADE in the accuracy of the benchmark functions *F*_3_ (a), *F*_4_ (b), and *F*_5_ (c). (2) Basic multimodal function *F*_6_–*F*_12_: the best performing algorithm is CoBiDE, which is better than MCDE on benchmark functions *F*_6_, *F*_8_, *F*_9_, and *F*_11_. Test results on *F*_7_ (d), *F*_10_ (e), and *F*_12_ (f) are worse than MCDE. Overall, the MCDE is similar to the CoBiDE on this type of benchmark function. (3) Extended multimodal function *F*_13_–*F*_14_ (g): the average error of the seven algorithms is at an order of magnitude, but the effects of JADE, CoDE, and MCDE are slightly better than the other four algorithms. (4) Complex function *F*_15_–*F*_25_ (h): MCDE is significantly better than JADE, jDE, CoDE, CoBiDE and LSHADE and slightly better than SaDE and EPSDE.

Based on the above four conclusions and Wilcoxon's test, it can be concluded that the MCDE shows significant effects in the four types of benchmark functions. Finally, the experimental results of the MCDE in *D* = 30 are better than those of JADE, jDE, SaDE, EPSDE, CoDE, CoBiDE, and LSHADE on 13, 13, 15, 18, 10, 13, and 11 benchmark functions, respectively, worse than other comparison algorithms on 3, 3, 5, 6, 5, 4, and 7 benchmark functions, and similar to other comparison algorithms on 9, 9, 5, 1, 10, 8, and 7 benchmark functions.

From Table 2, it can be seen that when Wilcoxon's detection is at *α* = 0.05 and *α* = 0.1, MCDE is more effective than JADE, jDE, SaDE, EPSDE, CoDE, CoBiDE, and LSHADE. According to Friedman's average ranking (*D* = 30) in Table 3, MCDE performed well in all types of benchmark functions and achieved the best ranking.

**Figure 3:**
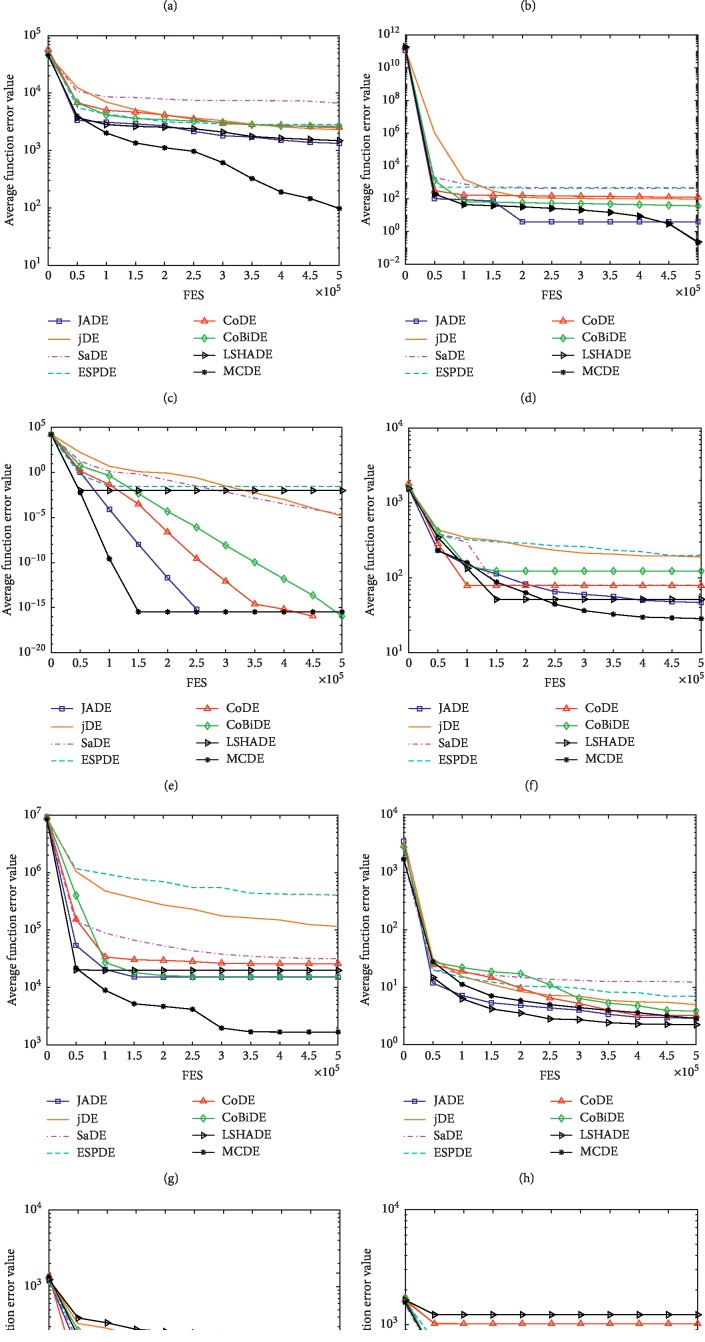
Evolution of the mean function error values derived from JADE, jDE, SaDE, CoDE, CoBiDE, LSHADE, and MCDE versus the number of FES on *F*_3_, *F*_4_, *F*_5_, *F*_6_, *F*_7_, *F*_10_, *F*_12_, *F*_13_, *F*_16_, and *F*_23_. (a) *D* = 50 *F*_3_. (b) *D* = 50 *F*_4_. (c) *D* = 50 *F*_5_. (d) *D* = 50 *F*_6_. (e) *D* = 50 *F*_7_. (f) *D* = 50 *F*_10_. (g) *D* = 50 *F*_12_. (h) *D* = 50 *F*_13_. (i) *D* = 50 *F*_16_. (j) *D* = 50 *F*_23_.

The experimental results of the above seven algorithms are shown in Table 4, where *D* = 50 and MaxFES = 500000. Based on the data given in Table 4 and Figure 3, we can draw the following conclusions. (1) Unimodal function *F*_1_–*F*_5_ : in *D* = 50, the effect of MCDE is higher than the other algorithms on *F*_4_ (b) and *F*_5_ (c). It shows that the search scope of mutation strategy is wider and the parameter-adaptive control performance is better. The test results of *F*_2_ and *F*_3_ are only inferior to the JADE and LSHADE. (2) Basic multimodal function *F*_6_–*F*_12_ : CoBiDE and LSHADE perform significantly better in this type of benchmark functions, whereas MCDE is slightly better than that on *F*_6_ (d), *F*_7_ (e), *F*_10_ (f), and *F*_12_ (g). (3) Extended multimodal function *F*_13_–*F*_14_ (h): the accuracy of MCDE is better than that of the other six algorithms, mainly because the proposed algorithm adopts a multiscale strategy to search for the best results. (4) Complex function *F*_15_–*F*_25_ (i) (j): such functions are the most complex problems on benchmark functions. There are 11 benchmark functions in all. The result of JADE, jDE, SaDE, CoDE, and LSHADE is not significant. The result of EPSDE is not stable, while MCDE, JADE, and CoBiDE are better than them.

Based on the above four conclusions and Wilcoxon's test, it can be concluded that MCDE shows significant effects in the four types of benchmark functions. Finally, the experimental results of the MCDE in *D* = 50 are better than the JADE, jDE, SaDE, EPSDE, CoDE, CoBiDE, and LSHADE on 18, 20, 21, 17, 22, 13, and 15 benchmark functions, worse than them on 4, 2, 2, 7, 2, 6, and 7 benchmark functions, and similar to them on 3, 3, 2, 1, 1, 6, and 3 benchmark functions, respectively.

From Table 5, it can be seen that when Wilcoxon's detection is at *α* = 0.05, MCDE is more effective than JADE, jDE, SaDE, EPSDE, CoDE, and LSHADE, and the *p* value of CoBiDE is 0.06. At *α* = 0.1, MCDE has significant differences from other algorithms. Based on Table 6 Friedman average ranking in *D* = 50, MCDE performed well in all types of benchmark functions and achieved the top ranking.

### 4.2. Comparison with Related Evolutionary Algorithms

To further evaluate MCDE, it is compared with CLPSO [47], CMA-ES [48], and GL-25 [25, 26]. CLPSO is a local version of the PSO, adopting a new learning strategy mechanism. CMA-ES adopts a covariance matrix adaptive mechanism and is mainly utilized to solve continuous optimization problems. GL-25 is a global and local real-coded genetic algorithm based on a new crossover operator. The experimental results of MCDE, CLPSO, CMA-ES, and GL-25 are shown in Table 7 at *D* = 30 and MaxFES = 300000. It can be concluded that MCDE has the most prominent effect on the unimodal function (*F*_2_–*F*_5_) and is smaller than the average error of other evolution algorithms. On the basic multimodal function *F*_10_ and *F*_12_, the result of MCDE is significantly better than other algorithms. On the extended multimodal functions and complex functions, most functions (*F*_14_, *F*_16_, *F*_17_, *F*_21_, *F*_23_, *F*_24_, and *F*_25_) have significant effects. Finally, the experimental results of MCDE in *D* = 30 are better than CLPSO, CMA-ES, and GL on 19, 15, and 21 benchmark functions, worse than those on 2, 5, and 1 benchmark functions, and similar to those on 4, 5, and 3 benchmark functions.

In this paper, the proposed algorithm is further compared with other evolution algorithms. From Table 8, it can be seen that when Wilcoxon's test detects *α* = 0.05 and *α* = 0.1, MCDE's *p* value is less than 0.05 and 0.1, and the effect is most significant. According to Table 9, the average ranking of Friedman under *D* = 30 shows that MCDE performs best on benchmark functions.

### 4.3. Runtime Comparison and Mechanism Comparison

In general, the running time of evolution algorithm contains the operating time of operator and the time of evaluating the fitness function. JADE, jDE, SaDE, EPSDE, CoDE, CoBiDE, and the proposed algorithms were run 25 times independently on 25 benchmark functions, and the average CPU time consumed was recorded. Set the parameters: MaxFES = 300000 and *D* = 30. To compare the average time, this paper determines the running speed of the algorithm by means of the mean CPU time ratio (AR) between the algorithms. AR > 1 shows that the algorithm runs slower than MCDE, and AR < 1 shows that the algorithm is faster than MCDE.

From the average AR in Table 10, it can be seen that its main range is [0.85, 13.41]. *j*DE runs at the fastest speed, and EPSDE runs at the slowest speed. The proposed algorithm is ranked third. The proposed algorithm is slower than jDE and JADE because multiscale strategies increase the search range but consume more time in mutation strategies.

**Figure 4:**
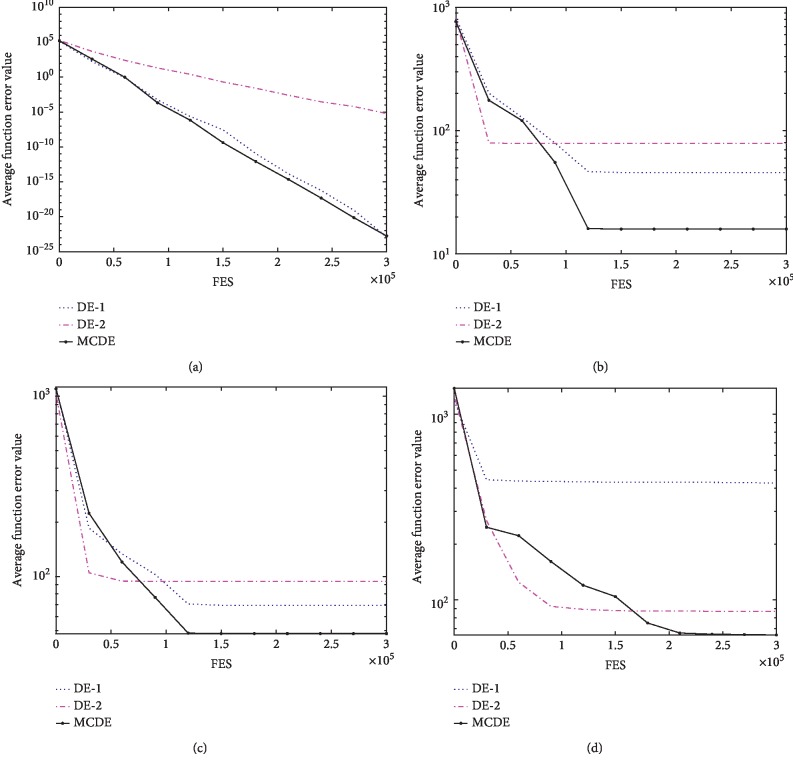
Evolution of the mean function error values derived from DE-1, DE-2, and MCDE versus the number of FES on *F*_4_, *F*_10_, *F*_15_, and *F*_17_. (a) The result of *F*_4_ under *D* = 30. (b) The result of *F*_10_ under *D* = 30. (c) the result of *F*_16_ under *D* = 30. (d) The result of *F*_17_ under *D* = 30.

By increasing the experiment with and without multigroup mechanism and covariance learning, it can be concluded from Table 11 and Figure 4 that the multiscale mechanism (DE-1) is outstanding in the unimodal function *F*_4_ (a). Covariance learning (DE-2) performs significantly in basic multimodal and complex functions with relatively complex structures in *F*_10_ (b), *F*_16_ (c), and *F*_17_ (d). The population structure is a multipopulation mechanism, and each subpopulation combines the corresponding mutation strategy to ensure the individual diversity in the evolutionary process. Then, the covariance learning establishes a proper rotation coordinate system for the crossover operation in the population. At the same time, adaptive control parameters are adopted to balance population survey and algorithm convergence.

## 5. Conclusions

MCDE introduces multiscale strategies, including local mutation strategies and global mutation strategies, to expand the population search scope. During evolution, the initial coordinate system is properly rotated by the covariance learning matrix to rotate the objective individual and the variant individual. During the covariance learning, the excellent crossover probability *CR* and the scaling factor *F* were inherited from the previous generation by the Lehmer mean and the power mean, respectively. The proposed algorithm is compared with JADE, jDE, SaDE, EPSDE, CoDE, CoBiDE, and LSHADE on the CEC 2005 benchmark function, and it can be seen that there are significant effects on the global optimization problem with *D* = 30 and *D* = 50. To further verify the algorithm, we compare it with other evolutionary algorithms such as CLPSO, CMA-ES, and GL-25 in *D* = 30 and discover it works best. In terms of running time, the proposed algorithm is in the upper part of the comparison algorithms. In summary, both the accuracy and the convergence speed have improved, so MCDE can be implemented.

## Figures and Tables

**Algorithm 1:**
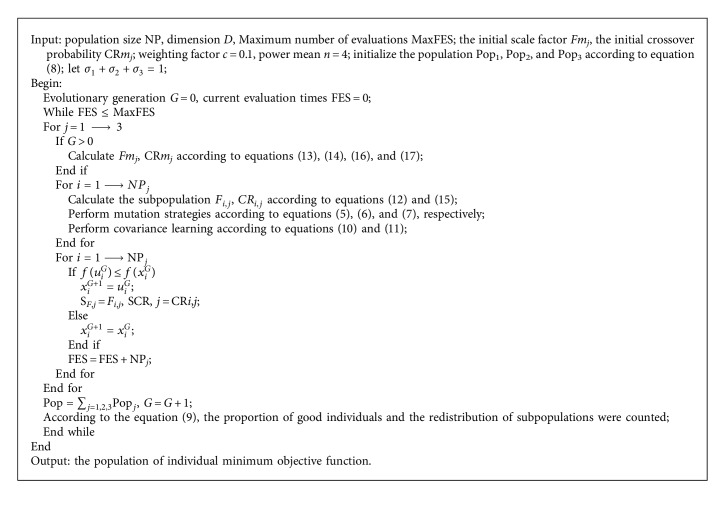
Pseudocode of the MCDE algorithm.

**Table 1:** Comparison results of MCDE with JADE, jDE, SaDE, EPSDE, CoDE, and CoBiDE (*D* = 30).

Function	JADE	jDE	SaDE	EPSDE	CoDE	CoBiDE	LSHADE	MCDE
*F*_1_	0.00*E* + 00 ± 0.00*E* + 00≈	0.00*E* + 00 ± 0.00*E* + 00≈	0.00*E* + 00 ± 0.00*E* + 00≈	0.00*E* + 00 ± 0.00*E* + 00≈	0.00*E* + 00 ± 0.00*E* + 00≈	0.00*E* + 00 ± 0.00*E* + 00≈	0.00*E* + 00 ± 0.00*E* + 00≈	0.00*E* + 00 ± 0.00*E* + 00
*F*_2_	1.49*E* − 28 ± 1.32*E* − 28+	1.06*E* − 06 ± 1.92*E* − 06−	1.21*E* − 05 ± 1.39*E* − 05−	4.25*E* − 10 ± 2.12*E* − 09−	2.39*E* − 15 ± 5.11*E* − 15−	1.52*E* − 12 ± 3.08*E* − 12−	3.08*E* − 29 ± 6.89*E* − 29+	8.52*E* − 28 ± 3.79*E* − 28
*F*_3_	8.36*E* + 03 ± 6.57*E* + 03−	1.67*E* + 05 ± 1.06*E* + 05−	3.71*E* + 05 ± 1.79*E* + 05−	1.37*E* + 06 ± 4.97*E* + 06−	1.27*E* + 05 ± 7.91*E* + 04−	8.86*E* + 04 ± 6.74*E* + 04−	7.70*E* + 03 ± 6.16*E* + 03−	3.82*E* − 12 ± 1.64*E* − 11
*F*_4_	1.88*E* − 15 ± 8.53*E* − 15−	2.06*E* − 02 ± 3.14*E* − 02−	1.14*E* + 01 ± 1.39*E* + 01−	2.30*E* + 05 ± 6.26*E* − 05−	5.24*E* − 03 ± 1.12*E* − 02−	1.35*E* − 03 ± 2.34*E* − 03−	5.05*E* − 15 ± 2.44*E* − 14−	7.12*E* − 22 ± 3.13*E* − 21
*F*_5_	3.86*E* − 08 ± 1.41*E* − 07−	3.27*E* + 03 ± 2.94*E* + 02−	2.19*E* + 03 ± 7.10*E* + 02−	9.15*E* + 02 ± 5.45*E* + 02−	5.52*E* + 02 ± 4.20*E* + 02−	1.04*E* + 02 ± 8.07*E* + 01−	7.09*E* − 10 ± 3.21*E* − 09−	4.54*E* − 10 ± 6.03*E* − 10
*F*_6_	1.73*E* + 01 ± 5.14*E* + 01−	1.96*E* + 01 ± 2.22*E* + 01−	5.61*E* + 01 ± 2.78*E* + 01−	3.18*E* − 01 ± 1.10*E* + 00−	3.18*E* − 01 ± 1.10*E* + 00−	3.73*E* − 02 ± 4.66*E* − 02+	1.59*E* − 01 ± 7.97*E* − 01+	3.18*E* − 01 ± 1.10*E* + 00
*F*_7_	8.17*E* − 03 ± 8.57*E* − 03−	1.18*E* − 02 ± 9.30*E* − 03−	1.19*E* − 02 ± 5.83*E* − 03−	1.22*E* − 02 ± 1.34*E* − 02−	6.99*E* − 03 ± 7.47*E* − 03−	3.25*E* − 03 ± 5.49*E* − 03−	7.88*E* − 03 ± 2.82*E* − 03−	1.67*E* − 03 ± 4.06*E* − 03
*F*_8_	2.08*E* + 01 ± 2.43*E* − 01≈	2.09*E* + 01 ± 3.91*E* − 02≈	2.09*E* + 01 ± 5.11*E* − 02≈	2.09*E* + 01 ± 6.24*E* − 02≈	2.02*E* + 01 ± 1.41*E* − 01+	2.07*E* + 01 ± 3.84*E* − 01+	2.04*E* + 01 ± 3.47*E* − 01+	2.09*E* + 01 ± 4.25*E* − 02
*F*_9_	0.00*E* + 00 ± 0.00*E* + 00+	0.00*E* + 00 ± 0.00*E* + 00+	0.00*E* + 00 ± 0.00*E* + 00+	0.00*E* + 00 ± 0.00*E* + 00+	0.00*E* + 00 ± 0.00*E* + 00+	0.00*E* + 00 ± 0.00*E* + 00+	0.00*E* + 00 ± 0.00*E* + 00+	2.56*E* − 07 ± 5.94*E* − 07
*F*_10_	2.37*E* + 01 ± 3.91*E* + 00−	5.45*E* + 01 ± 9.77*E* + 00−	3.86*E* + 01 ± 8.61*E* + 01−	6.14*E* + 01 ± 9.02*E* + 00−	4.23*E* + 01 ± 1.12*E* + 01−	4.28*E* + 01 ± 1.44*E* + 01−	1.53*E* + 01 ± 2.74*E* + 00+	2.23*E* + 01 ± 6.39*E* + 00
*F*_11_	2.55*E* + 01 ± 1.62*E* + 00−	2.82*E* + 01 ± 1.71*E* + 00−	1.97*E* + 01 ± 7.77*E* + 00−	3.57*E* + 01 ± 2.87*E* + 00−	1.31*E* + 01 ± 3.10*E* + 00+	6.05*E* + 00 ± 2.24*E* + 00+	2.68*E* + 01 ± 1.69*E* + 00−	1.45*E* + 01 ± 7.73*e* + 00
*F*_12_	5.88*E* + 03 ± 3.63*E* + 03−	1.17*E* + 04 ± 9.44*E* + 03−	5.15*E* + 03 ± 4.33*E* + 03−	5.75*E* + 04 ± 1.05*E* + 04−	2.81*E* + 03 ± 3.19*E* + 03−	3.86*E* + 03 ± 5.02*E* + 03−	2.44*E* + 03 ± 2.44*E* + 03−	1.71*E* + 03 ± 2.33*E* + 03
*F*_13_	1.45*E* + 00 ± 1.16*E* − 01+	1.71*E* + 00 ± 1.37*E* − 01+	4.55*E* + 00 ± 3.10*E* − 01−	2.52*E* + 00 ± 1.77*E* − 01−	1.61*E* + 00 ± 4.13*E* − 01+	2.69*E* + 00 ± 99.2*E* − 01−	1.17*E* + 00 ± 1.11*E* − 01+	1.78*E* + 00 ± 2.04*E* − 01
*F*_14_	1.23*E* + 01 ± 3.21*E* − 01≈	1.29*E* + 01 ± 2.88*E* − 01−	1.28*E* + 01 ± 2.24*E* − 01−	1.34*E* + 01 ± 1.98*E* − 01−	1.23*E* + 01 ± 5.90*E* − 01≈	1.22*E* + 01 ± 5.23*E* − 01≈	1.23*E* + 01 ± 4.08*E* − 01≈	1.23*E* + 01 ± 2.53*E* − 01
*F*_15_	3.26*E* + 02 ± 1.27*E* + 02+	3.56*E* + 02 ± 6.46*E* + 01+	3.84*E* + 02 ± 7.48*E* + 01+	2.36*E* + 02 ± 4.25*E* + 01+	4.00*E* + 02 ± 5.00*E* + 01≈	4.12*E* + 02 ± 6.00*E* + 01−	3.56*E* + 02 ± 7.11*E* + 01+	4.00*E* + 02 ± 1.15*E* + 02
*F*_16_	9.14*E* + 01 ± 9.89*E* + 01−	8.04*E* + 01 ± 2.51*E* + 01−	5.99*E* + 01 ± 2.16*E* + 01−	1.18*E* + 02 ± 8.78*E* + 01−	6.89*E* + 01 ± 2.23*E* + 01+	6.50*E* + 01 ± 1.26*E* + 01−	9.07*E* + 01 ± 1.32*E* + 02−	5.48*E* + 01 ± 2.92*E* + 01
*F*_17_	1.44*E* + 02 ± 1.34*E* + 02−	1.40*E* + 02 ± 3.24*E* + 01−	8.38*E* + 01 ± 7.68*E* + 01−	1.74*E* + 02 ± 7.24*E* + 01−	7.24*E* + 01 ± 2.54*E* + 01−	8.03*E* + 01 ± 7.42*e* + 01−	1.08*E* + 02 ± 1.11*E* + 02−	6.41*E* + 01 ± 7.58*E* + 01
*F*_18_	9.04*E* + 02 ± 7.97*E* − 01≈	9.04*E* + 02 ± 9.87*E* − 01≈	8.69*E* + 02 ± 5.78*E* + 01+	8.16*E* + 02 ± 5.16*E* − 01+	9.04*E* + 02 ± 1.31*E* + 00≈	9.04*E* + 02 ± 7.16*E* − 01≈	9.03*E* + 02 ± 6.22*E* − 01≈	9.03*E* + 02 ± 5.59*E* − 01
*F*_19_	9.04*E* + 02 ± 1.02*E* + 00≈	9.04*E* + 02 ± 9.35*E* − 01≈	8.51*E* + 02 ± 5.76*E* + 01+	8.16*E* + 02 ± 5.59*E* − 01+	9.04*E* + 02 ± 8.03*E* − 01≈	9.04*E* + 02 ± 8.61*E* − 01≈	9.03*E* + 02 ± 7.09*E* − 01≈	9.03*E* + 02 ± 2.01*E* − 01
*F*_20_	9.04*E* + 02 ± 9.61*E* − 01≈	9.04*E* + 02 ± 1.04*E* + 00≈	8.51*E* + 02 ± 5.77*E* + 02+	8.16*E* + 02 ± 4.03*E* − 01+	9.04*E* + 02 ± 7.11*E* − 01≈	9.04*E* + 02 ± 7.38*E* − 01≈	9.03*E* + 02 ± 7.21*E* − 01≈	9.03*E* + 02 ± 2.40*E* − 01
*F*_21_	5.00*E* + 02 ± 7.33*E* − 14≈	5.00*E* + 02 ± 8.83*E* − 14≈	5.00*E* + 02 ± 9.84*E* − 14≈	8.58*E* + 02 ± 1.37*E* + 02−	5.00*E* + 02 ± 9.84*E* − 14≈	5.00*E* + 02 ± 7.46*E* − 14≈	5.00*E* + 02 ± 1.25*E* − 13≈	5.00*E* + 02 ± 6.76*E* − 14
*F*_22_	8.63*E* + 02 ± 2.06*E* + 01≈	8.81*E* + 02 ± 1.46*E* + 01≈	9.16*E* + 02 ± 1.54*E* + 01−	5.04*E* + 02 ± 3.63*E* + 00+	8.62*E* + 02 ± 2.22*E* + 01≈	8.69*E* + 02 ± 3.42*E* + 01≈	8.75*E* + 02 ± 1.69*E* + 01−	8.69*E* + 02 ± 1.92*E* + 01
*F*_23_	5.66*E* + 02 ± 1.12*E* + 02−	5.34*E* + 02 ± 2.14*E* − 04≈	5.34*E* + 02 ± 2.74*E* − 03≈	8.66*E* + 02 ± 1.45*E* + 00−	5.34*E* + 02 ± 4.13*E* − 04≈	5.34*E* + 02 ± 2.43*E* − 04≈	5.34*E* + 02 ± 2.28*E* − 13≈	5.34*E* + 02 ± 3.40*E* − 13
*F*_24_	2.00*E* + 02 ± 2.90*E* − 14≈	2.00*E* + 02 ± 2.90*E* − 14≈	2.00*E* + 02 ± 2.90*E* − 14≈	2.11*E* + 02 ± 1.16*E* + 00−	2.00*E* + 02 ± 2.90*E* − 14≈	2.00*E* + 02 ± 2.90*E* − 14≈	2.29*E* + 02 ± 1.48*E* + 02−	2.00*E* + 02 ± 2.90*E* − 14
*F*_25_	2.11*E* + 02 ± 8.77*E* − 01−	2.11*E* + 02 ± 7.33*E* + 02−	2.11*E* + 02 ± 1.23*E* + 00−	2.11*E* + 02 ± 2.52*E* + 00−	2.10*E* + 02 ± 7.22*E* − 01−	2.10*E* + 02 ± 8.03*E* − 01−	2.11*E* + 02 ± 7.91*E* − 01−	2.09*E* + 02 ± 2.84*E* − 01
−/+/≈	**13/3/9**	**13/3/9**	**15/5/5**	**18/6/1**	**10/5/10**	**13/4/8**	**11/7/7**	

**Table 2:** Wilcoxon's detection (*D* = 30).

Algorithm	*R* ^+^	*R* ^−^	*p* value	*α* = 0.05	*α* = 0.1
MCDE vs JADE	249.0	76.0	0.017653	Yes	Yes
MCDE vs jDE	262.0	38.0	0.001111	Yes	Yes
MCDE vs SaDE	224.0	76.0	0.032663	Yes	Yes
MCDE vs EPSDE	219.5	80.5	0.027602	Yes	Yes
MCDE vs CoDE	225.5	74.5	0.027602	Yes	Yes
MCDE vs CoBiDE	243.0	57.0	0.006725	Yes	Yes
MCDE vs LSHADE	196.5	103.5	0.19244	No	No

**Table 3:** Friedman's average ranking (*D* = 30).

Algorithm	Ranking	Final rank
JADE	4.48	5
jDE	5.76	7
SaDE	5.08	6
EPSDE	5.68	8
CoDE	3.92	3
CoBiDE	4.22	4
LSHADE	3.62	2
MCDE	3.24	1

**Table 4:** Comparison results of MCDE with JADE, jDE, SaDE, EPSDE, CoDE, and CoBiDE (*D* = 50).

Function	JADE	jDE	SaDE	EPSDE	CoDE	CoBiDE	LSHADE	MCDE
*F*_1_	0.00*E* + 00 ± 0.00*E* + 00≈	0.00*E* + 00 ± 0.00*E* + 00≈	0.00*E* + 00 ± 0.00*E* + 00≈	5.25*E* − 29 ± 1.28*E* − 28−	8.07*E* − 30 ± 4.04*E* − 29−	0.00*E* + 00 ± 0.00*E* + 00≈	0.00*E* + 00 ± 0.00*E* + 00≈	0.00*E* + 00 ± 0.00*E* + 00
*F*_2_	3.57*E* − 27 ± 1.67*E* − 27+	2.22*E* − 02 ± 3.21*E* − 02−	6.55*E* − 02 ± 4.34*E* − 02−	5.77*E* − 23 ± 8.87*E* − 23+	5.87*E* − 09 ± 6.51*E* − 09−	1.03*E* − 06 ± 1.08*E* − 06−	6.85*E* − 27 ± 7.92*E* − 27+	5.76*E* − 13 ± 1.01*E* − 12
*F*_3_	1.41*E* + 05 ± 6.61*E* + 03−	4.48*E* + 05 ± 2.21*E* + 05−	8.01*E* + 05 ± 5.44*E* + 05−	7.82*E* + 06 ± 1.35*E* + 07−	3.22*E* + 05 ± 8.66*E* + 04−	2.31*E* + 05 ± 7.87*E* + 04−	1.97*E* + 04 ± 1.07*E* + 04+	4.31*E* + 04 ± 3.02*E* + 04
*F*_4_	2.79*E* + 00 ± 1.26*E* + 01−	3.88*E* + 02 ± 3.13*E* + 02−	7.28*E* + 02 ± 4.56*E* + 02−	3.42*E* + 03 ± 3.51*E* + 04−	4.98*E* + 02 ± 5.78*E* + 02−	1.52*E* + 02 ± 9.61*E* + 01−	2.24*E* + 01 ± 5.73*E* + 01−	8.51*E* − 01 ± 8.82*E* − 01
*F*_5_	1.45*E* + 03 ± 4.38*E* + 02−	3.65*E* + 03 ± 6.29*E* + 02−	8.34*E* + 03 ± 1.29*E* + 03−	4.66*E* + 03 ± 8.85*E* + 02−	3.60*E* + 03 ± 5.69*E* + 02−	2.71*E* + 03 ± 6.16*E* + 02−	1.12*E* + 03 ± 4.72*E* + 02−	2.09*E* + 02 ± 4.11*E* + 02
*F*_6_	7.71*E* + 00 ± 3.03*E* + 01−	4.43*E* + 02 ± 2.78*E* + 01−	4.39*E* + 02 ± 2.52*E* + 01−	1.43*E* + 02 ± 1.95*E* + 00−	1.23*E* + 02 ± 2.12*E* + 00−	2.36*E* + 01 ± 1.88*E* + 01−	7.37*E* − 01 ± 1.49*E* + 00−	7.21*E* − 01 ± 1.46*E* + 00
*F*_7_	7.57*E* − 03 ± 1.09*E* − 02−	2.85*E* − 03 ± 6.10*E* − 03+	9.23*E* − 03 ± 5.45*E* − 02−	1.08*E* − 02 ± 1.79*E* − 02−	6.54*E* − 03 ± 9.36*E* − 03−	4.01*E* − 03 ± 3.09*E* − 03−	2.16*E* − 03 ± 4.04*E* − 03+	3.05*E* − 03 ± 6.32*E* − 03
*F*_8_	2.11*E* + 01 ± 5.93*E* − 02≈	2.11*E* + 01 ± 3.72*E* − 02≈	2.11*E* + 01 ± 4.33*E* − 02≈	2.11*E* + 01 ± 3.35*E* − 02≈	2.01*E* + 01 ± 1.09*E* − 01+	2.08*E* + 01 ± 4.45*E* − 01+	2.07*E* + 01 ± 2.89*E* − 01+	2.11*E* + 01 ± 5.06*E* − 02
*F*_9_	0.00*E* + 00 ± 0.00*E* + 00+	0.00*E* + 00 ± 0.00*E* + 00+	9.94*E* − 01 ± 2.21*E* − 01−	6.39*E* − 16 ± 1.01*E* − 15+	2.38*E* + 00 ± 4.33*E* − 01−	3.21*E* − 13 ± 9.03*E* − 13+	7.11*E* − 17 ± 3.55*E* − 16+	9.55*E* − 01 ± 2.92*E* + 00
*F*_10_	6.55*E* + 01 ± 6.93*E* + 00−	9.98*E* + 01 ± 1.32*E* + 01−	1.14*E* + 02 ± 1.54*E* + 01−	1.54*E* + 02 ± 2.53*E* + 01−	8.29*E* + 01 ± 1.93*E* + 01−	9.24*E* + 01 ± 2.04*E* + 01−	4.60*E* + 01 ± 5.30*E* + 00−	4.31*E* + 01 ± 1.15*E* + 01
*F*_11_	5.24*E* + 01 ± 2.15*E* + 00−	5.42*E* + 01 ± 2.10*E* + 00−	4.45*E* + 01 ± 1.89*E* + 00−	7.04*E* + 01 ± 3.21*E* + 00−	3.14*E* + 01 ± 5.35*E* + 00−	1.80*E* + 01 ± 4.49*E* + 00+	5.39*E* + 01 ± 3.26*E* + 00−	3.03*E* + 01 ± 8.38*E* + 00
*F*_12_	1.54*E* + 04 ± 1.27*E* + 04−	1.57*E* + 04 ± 1.54*E* + 04−	5.65*E* + 04 ± 2.04*E* + 04−	3.16*E* + 05 ± 3.85*E* + 04−	1.54*E* + 04 ± 1.73*E* + 04−	1.35*E* + 04 ± 1.22*E* + 04−	1.21*E* + 04 ± 1.04*E* + 04−	1.16*E* + 04 ± 1.01*E* + 04
*F*_13_	2.78*E* + 00 ± 1.99*E* − 01−	2.94*E* + 00 ± 2.41*E* − 01−	7.27*E* + 00 ± 7.34*E* − 01−	6.17*E* + 00 ± 6.03*E* − 01−	3.23*E* + 00 ± 4.15*E* − 01−	4.28*E* + 00 ± 1.32*E* + 00−	2.14*E* + 00 ± 1.03*E* − 01+	2.72*E* + 00 ± 4.54*E* − 01
*F*_14_	2.16*E* + 01 ± 4.76*E* − 01−	2.25*E* + 01 ± 2.26*E* − 01−	2.23*E* + 01 ± 2.42*E* − 01−	2.34*E* + 01 ± 2.63*E* − 01−	2.19*E* + 01 ± 4.39*E* − 01−	2.20*E* + 01 ± 3.87*E* − 01−	2.18*E* + 01 ± 3.12*E* − 01−	2.12*E* + 01 ± 3.24*E* − 01
*F*_15_	3.77*E* + 02 ± 9.03*E* + 01−	3.62*E* + 02 ± 1.21*E* + 02+	3.86*E* + 02 ± 7.62*E* + 01−	2.64*E* + 02 ± 6.45*E* + 01+	3.88*E* + 02 ± 6.00*E* + 01−	3.92*E* + 02 ± 4.00*E* + 01−	2.97*E* + 02 ± 9.67*E* + 01+	3.68*E* + 02 ± 6.23*E* + 01
*F*_16_	8.42*E* + 01 ± 7.92*E* + 01−	8.35*E* + 01 ± 1.03*E* + 01−	8.78*E* + 01 ± 6.57*E* + 01−	1.50*E* + 02 ± 4.25*E* + 01−	9.35*E* + 01 ± 7.01*E* + 01−	7.64*E* + 01 ± 2.11*E* + 01−	9.82*E* + 02 ± 1.35 + 02−	7.56*E* + 01 ± 1.01*E* + 02
*F*_17_	9.39*E* + 01 ± 2.64*E* + 01−	1.81*E* + 02 ± 2.31*E* + 01−	9.81*E* + 01 ± 1.01*E* + 02−	2.38*E* + 02 ± 7.01*E* + 01−	7.21*E* + 01 ± 2.58*E* + 01+	7.62*E* + 01 ± 1.73*E* + 01+	1.18*E* + 02 ± 1.08*E* + 02−	8.84*E* + 01 ± 1.26*E* + 02
*F*_18_	9.21*E* + 02 ± 4.38*E* + 00−	9.20*E* + 02 ± 3.35*E* + 00−	9.78*E* + 02 ± 8.37*E* + 01−	8.53*E* + 02 ± 2.42*E* + 01+	9.21*E* + 02 ± 5.42*E* + 00−	9.12*E* + 02 ± 2.39*E* + 01≈	9.18*E* + 02 ± 4.25*E* + 00−	9.14*E* + 02 ± 9.46*E* + 00
*F*_19_	9.19*E* + 02 ± 1.07*E* + 01−	9.20*E* + 02 ± 2.88*E* + 00−	9.78*E* + 02 ± 7.81*E* + 01−	8.59*E* + 02 ± 1.54*E* + 01+	9.21*E* + 02 ± 4.64*E* + 00−	9.14*E* + 02 ± 2.39*E* + 01≈	9.18*E* + 02 ± 6.62*E* + 00−	9.15*E* + 02 ± 2.90*E* + 00
*F*_20_	9.21*E* + 02 ± 3.38*E* + 00−	9.20*E* + 02 ± 3.14*E* + 00−	9.55*E* + 02 ± 3.54*E* + 01−	8.56*E* + 02 ± 3.03*E* + 00+	9.11*E* + 02 ± 3.38*E* + 01−	9.18*E* + 02 ± 3.40*E* + 00≈	9.17*E* + 02 ± 7.36*E* + 00≈	9.16*E* + 02 ± 3.94*E* + 00
*F*_21_	5.52*E* + 02 ± 1.49*E* + 02+	7.21*E* + 02 ± 2.55*E* + 02−	5.66*E* + 02 ± 2.32*E* + 02+	7.29*E* + 02 ± 2.82*E* + 00−	6.83*E* + 02 ± 2.49*E* + 02−	5.40*E* + 02 ± 1.41*E* + 02+	6.82*E* + 02 ± 2.48*E* + 02−	6.21*E* + 02 ± 2.21*E* + 02
*F*_22_	9.05*E* + 02 ± 2.48*E* + 01−	9.05*E* + 02 ± 1.23*E* + 00−	9.82*E* + 02 ± 8.21*E* + 01−	5.00*E* + 02 ± 6.61*E* − 02+	9.01*E* + 02 ± 2.18*E* + 01−	8.84*E* + 02 ± 2.53*E* + 01+	8.97*E* + 02 ± 1.94*E* + 01≈	8.97*E* + 02 ± 1.65*E* + 01
*F*_23_	5.82*E* + 02 ± 1.31*E* + 02+	8.60*E* + 02 ± 2.25*E* + 02−	5.98*E* + 02 ± 7.29*E* + 00+	7.33*E* + 02 ± 4.48*E* + 00−	7.10*E* + 02 ± 2.33*E* + 02−	6.91*E* + 02 ± 2.25*E* + 02≈	8.04*E* + 02 ± 2.40*E* + 02−	6.90*E* + 02 ± 2.25*E* + 02
*F*_24_	2.00*E* + 02 ± 1.49*E* − 12≈	2.00*E* + 02 ± 1.62*E* − 12≈	2.89*E* + 02 ± 6.55*E* + 01−	2.38*E* + 02 ± 1.34*E* + 01−	2.00*E* + 02 ± 5.81*E* − 14≈	2.00*E* + 02 ± 1.08*E* − 12≈	2.32*E* + 02 ± 1.57*E* + 02−	2.00*E* + 02 ± 1.66*E* − 12
*F*_25_	2.18*E* + 02 ± 1.71*E* + 00−	2.16*E* + 02 ± 1.41*E* + 00−	2.24*E* + 02 ± 1.25*E* + 01−	2.47*E* + 02 ± 1.87*E* + 01−	2.17*E* + 02 ± 1.97*E* + 00−	2.15*E* + 02 ± 1.46*E* + 00−	2.18*E* + 02 ± 1.69*E* + 00−	2.14*E* + 02 ± 7.36*E* − 01
−/+/≈	**18/4/3**	**20/2/3**	**21/2/2**	**17/7/1**	**22/2/1**	**13/6/6**	**15/7/3**	

**Table 5:** Wilcoxon's detection (*D* = 50).

Algorithm	*R* ^+^	*R* ^−^	*p* value	*α* = 0.05	*α* = 0.1
MCDE vs JADE	267.5	32.5	0.000716	Yes	Yes
MCDE vs jDE	278.0	22.0	0.000229	Yes	Yes
MCDE vs SaDE	303.5	21.5	0.00014	Yes	Yes
MCDE vs EPSDE	223.0	77	0.035729	Yes	Yes
MCDE vs CoDE	269.0	31.0	0.000611	Yes	Yes
MCDE vs CoBiDE	214.0	86.0	0.060605	No	Yes
MCDE vs LSHADE	250.5	74.5	0.016642	Yes	Yes

**Table 6:** Friedman's average ranking (*D* = 50).

Algorithm	Ranking	Final rank
JADE	3.98	4
jDE	5.36	6
SaDE	6.46	8
EPSDE	5.64	7
CoDE	4.88	5
CoBiDE	3.54	2
LSHADE	3.56	3
MCDE	2.58	1

**Table 7:** Comparison results of MCDE with CLPSO, CMA-ES, and GL-25 (*D* = 30).

Function		CLPSO	CMA-ES	GL-25	MCDE
Unimodal function	*F*_1_	0.00*E* + 00 ± 0.00*E* + 00≈	1.58*E* − 25 ± 3.35*E* − 26−	5.60*E* − 27 ± 1.76*E* − 26−	0.00*E* + 00 ± 0.00*E* + 00
	*F*_2_	8.40*E* + 02 ± 1.90*E* + 02−	1.12*E* − 24 ± 2.93*E* − 25−	4.04*E* + 01 ± 6.28*E* + 01−	8.52*E* − 28 ± 3.79*E* − 28
	*F*_3_	1.42*E* + 07 ± 4.19*E* + 06−	5.54*E* − 21 ± 1.69*E* − 21+	2.19*E* + 06 ± 1.08*E* + 06−	3.82*E* − 12 ± 1.64*E* − 11
	*F*_4_	6.99*E* + 03 ± 1.73*E* + 03−	9.15*E* + 05 ± 2.16*E* + 06−	9.07*E* + 02 ± 4.25*E* + 02−	7.12*E* − 22 ± 3.13*E* − 21
	*F*_5_	3.86*E* + 03 ± 4.35*E* + 02−	2.77*E* − 10 ± 5.04*E* − 11+	2.51*E* + 03 ± 1.96*E* + 02−	4.54*E* − 10 ± 6.03*E* − 10

Basic multimodal function	*F*_6_	4.16*E* + 00 ± 3.48*E* + 00−	4.78*E* − 01 ± 1.32*E* + 00−	2.15*E* + 01 ± 1.17*E* + 00−	3.18*E* − 01 ± 1.10*E* + 00
	*F*_7_	4.51*E* − 01 ± 8.47*E* − 02−	1.82*E* − 03 ± 4.33*E* − 03−	2.78*E* − 02 ± 3.62*E* − 02−	1.67*E* − 03 ± 4.06*E* − 03
	*F*_8_	2.09*E* + 01 ± 4.41*E* − 02−	2.03*E* + 01 ± 5.72*E* − 01+	2.09*E* + 01 ± 5.94*E* − 02−	2.09*E* + 01 ± 4.25*E* − 02
	*F*_9_	0.00*E* + 00 ± 0.00*E* + 00+	4.45*E* + 02 ± 7.12*E* + 01−	2.45*E* + 01 ± 7.35*E* + 00−	2.56*E* − 07 ± 5.94*E* − 07
	*F*_10_	1.04*E* + 02 ± 1.53*E* + 01−	4.63*E* + 01 ± 1.16*E* + 01−	1.42*E* + 02 ± 6.45*E* + 01−	2.23*E* + 01 ± 6.39*E* + 00
	*F*_11_	2.60*E* + 01 ± 1.63*E* + 00−	7.11*E* + 00 ± 2.14*E* + 00+	3.27*E* + 01 ± 7.79*E* + 00−	1.45*E* + 01 ± 7.73*E* + 00
	*F*_12_	1.79*E* + 04 ± 5.24*E* + 03−	1.26*E* + 04 ± 1.74*E* + 04−	6.53*E* + 04 ± 4.69*E* + 04−	1.71*E* + 03 ± 2.33*E* + 03

Expanded multimodal function	*F*_13_	2.06*E* + 00 ± 2.15*E* − 01−	3.43*E* + 00 ± 7.60*E* − 01−	6.23*E* + 00 ± 4.88*E* + 00−	1.78*E* + 00 ± 2.04*E* − 01
	*F*_14_	1.28*E* + 01 ± 2.48*E* − 01−	1.47*E* + 01 ± 3.31*E* − 01−	1.31*E* + 01 ± 1.84*E* − 01−	1.23*E* + 01 ± 2.53*E* − 01

Hybrid composition function	*F*_15_	5.77*E* + 01 ± 2.76*E* + 01−	5.55*E* + 02 ± 3.32*E* + 02−	3.04*E* + 02 ± 1.99*E* + 01+	4.00*E* + 02 ± 1.15*E* + 02
	*F*_16_	1.74*E* + 02 ± 2.82*E* + 01−	2.98*E* + 02 ± 2.08*E* + 02−	1.32*E* + 02 ± 7.60*E* + 01−	5.48*E* + 01 ± 2.92*E* + 01
	*F*_17_	2.46*E* + 02 ± 4.81*E* + 01−	4.43*E* + 02 ± 3.34*E* + 02−	1.61*E* + 02 ± 6.80*E* + 01−	6.41*E* + 01 ± 7.58*E* + 01
	*F*_18_	9.13*E* + 02 ± 1.42*E* + 00−	9.04*E* + 02 ± 3.01*E* − 01≈	9.07*E* + 02 ± 1.48*E* + 00−	9.03*E* + 02 ± 5.59*E* − 01
	*F*_19_	9.14*E* + 02 ± 1.45*E* + 00−	9.16*E* + 02 ± 6.03*E* + 01−	9.06*E* + 02 ± 1.24*E* + 00−	9.03*E* + 02 ± 2.01*E* − 01
	*F*_20_	9.14*E* + 02 ± 3.62*E* + 00−	9.04*E* + 02 ± 2.71*E* − 01≈	9.07*E* + 02 ± 1.35*E* + 00−	9.03*E* + 02 ± 2.40*E* − 01
	*F*_21_	5.00*E* + 02 ± 3.39*E* − 13≈	5.00*E* + 02 ± 2.68*E* − 12≈	5.00*E* + 02 ± 4.83*E* − 13≈	5.00*E* + 02 ± 6.76*E* − 14
	*F*_22_	9.72*E* + 02 ± 1.20*E* + 01−	8.26*E* + 02 ± 1.46*E* + 01+	9.28*E* + 02 ± 7.04*E* + 01−	8.69*E* + 02 ± 1.92*E* + 01
	*F*_23_	5.34*E* + 02 ± 2.19*E* − 04≈	5.36*E* + 02 ± 5.44*E* + 00≈	5.34*E* + 02 ± 4.66*E* − 04≈	5.34*E* + 02 ± 3.40*E* − 13
	*F*_24_	2.00*E* + 02 ± 1.49*E* − 12≈	2.12*E* + 02 ± 6.00*E* + 01−	2.00*E* + 02 ± 5.52*E* − 11≈	2.00*E* + 02 ± 2.90*E* − 14
	*F*_25_	2.00*E* + 02 ± 1.96*E* + 00+	2.07*E* + 02 ± 6.07*E* + 00≈	2.17*E* + 02 ± 1.36*E* − 01−	2.09*E* + 02 ± 2.84*E* − 01

−/+/≈		**19/2/4**	**15/5/5**	**21/1/3**	

**Table 8:** Wilcoxon's detection with evolution algorithm.

Algorithm	*R* ^+^	*R* ^−^	*p* value	*α* = 0.05	*α* = 0.1
MCDE vs CLPSO	261.0	39.0	0.001388	Yes	Yes
MCDE vs CMA-ES	241.0	59.0	0.008699	Yes	Yes
MCDE vs GL	301.0	24.0	0.000175	Yes	Yes

**Table 9:** Friedman average ranking with evolution algorithm.

Algorithm	Ranking	Final rank
CLPSO	2.86	3
CMA-ES	2.6	2
GL	2.92	4
MCDE	1.62	1

**Table 10:** Average CPU time for MCDE, JADE, *j*DE, SaDE, EPSDE, CoDE, and CoBiDE (*D* = 30).

Function	JADE	jDE	SaDE	EPSDE	CoDE	CoBiDE	MCDE
*F*_1_	2.20	1.77	38.72	73.93	6.27	3.78	2.54
*F*_2_	2.29	2.04	35.61	76.71	6.52	4.18	2.88
*F*_3_	2.53	2.19	35.34	72.37	7.45	4.25	2.90
*F*_4_	2.34	2.14	31.52	73.96	6.66	4.28	3.01
*F*_5_	2.63	2.41	35.49	76.41	7.29	4.19	3.32
*F*_6_	2.15	1.87	33.71	79.66	6.34	3.87	2.79
*F*_7_	2.34	2.24	30.73	74.79	6.75	4.05	2.70
*F*_8_	2.82	2.72	33.82	71.86	8.96	4.74	3.18
*F*_9_	2.37	2.05	34.66	73.91	6.95	4.01	2.85
*F*_10_	2.65	2.50	32.28	72.04	7.81	4.30	2.94
*F*_11_	28.84	28.14	103.99	98.35	32.21	31.09	29.02
*F*_12_	10.30	10.44	65.05	81.98	14.53	12.72	10.76
*F*_13_	2.47	2.13	31.88	71.55	6.78	4.49	3.02
*F*_14_	3.16	2.88	34.24	72.24	8.75	5.09	3.65
*F*_15_	76.01	73.49	202.83	150.91	77.95	85.29	75.28
*F*_16_	73.83	72.74	202.17	144.18	76.25	82.40	75.63
*F*_17_	73.66	72.98	204.50	143.44	78.80	83.14	77.24
*F*_18_	82.29	75.51	211.46	150.07	80.43	86.19	79.29
*F*_19_	81.26	75.79	211.67	151.88	81.01	86.29	78.97
*F*_20_	84.06	75.81	211.70	150.82	80.46	86.50	79.04
*F*_21_	81.41	73.25	211.90	154.60	78.09	85.74	77.24
*F*_22_	99.15	91.68	256.54	196.01	96.17	103.81	92.63
*F*_23_	79.61	73.79	216.39	171.24	80.19	87.85	75.63
*F*_24_	56.19	52.84	150.57	145.81	56.74	64.01	54.80
*F*_25_	59.55	56.30	154.85	143.67	59.98	67.57	58.39
Average AR	**0.93**	**0.85**	**6.99**	**13.41**	**1.71**	**1.27**	

**Table 11:** Comparison results of MCDE with DE-1 and DE-2 (*D* = 30).

Function	DE-1	DE-2	MCDE
*F*_1_	0.00*E* + 00 ± 0.00*E* + 00≈	0.00*E* + 00 ± 0.00*e* + 00≈	0.00*E* + 00 ± 0.00*E* + 00
*F*_2_	6.89*E* − 28 ± 8.87*E* − 28≈	2.24*E* − 14 ± 2.68*E* − 13−	8.52*E* − 28 ± 3.79*E* − 28
*F*_3_	1.98*E* − 12 ± 7.27*E* − 11≈	1.23*E* + 05 ± 8.34*E* + 04−	3.82*E* − 12 ± 1.64*E* − 11
*F*_4_	2.09*E* − 24 ± 3.79*E* − 24+	1.97*E* − 04 ± 3.69*E* − 04−	7.12*E* − 22 ± 3.13*E* − 21
*F*_5_	1.26*E* − 09 ± 1.89*E* − 09−	1.91*E* + 01 ± 6.15*E* + 01−	4.54*E* − 10 ± 6.03*E* − 10
*F*_6_	4.78*E* − 01 ± 1.32*E* + 00≈	1.84*E* − 10 ± 4.18*E* − 10+	3.18*E* − 01 ± 1.10*E* + 00
*F*_7_	9.86*E* − 04 ± 2.75*E* − 03+	5.80*E* − 03 ± 1.05*E* − 02−	1.67*E* − 03 ± 4.06*E* − 03
*F*_8_	2.09*E* + 01 ± 6.90*E* − 02≈	2.00*E* + 01 ± 1.58*E* − 02+	2.09*E* + 01 ± 4.25*E* − 02
*F*_9_	3.43*E* − 13 ± 3.53*E* − 13+	0.00*E* + 00 ± 0.00*e* + 00+	2.56*E* − 07 ± 5.94*E* − 07
*F*_10_	3.02*E* + 01 ± 9.89*E* + 00−	5.18*E* + 01 ± 1.23*E* + 01−	2.23*E* + 01 ± 6.39*E* + 00
*F*_11_	1.74*E* + 01 ± 6.49*E* + 00−	9.16*E* + 00 ± 3.91*E* + 00−	1.45*E* + 01 ± 7.73*e* + 00
*F*_12_	3.89*E* + 03 ± 1.08*E* + 03−	4.75*E* + 03 ± 6.16*E* + 03−	1.71*E* + 03 ± 2.33*E* + 03
*F*_13_	2.01*E* + 00 ± 2.49*E* − 01−	1.95*E* + 00 ± 5.26*E* − 01−	1.78*E* + 00 ± 2.04*E* − 01
*F*_14_	1.21*E* + 01 ± 3.12*E* − 01≈	1.28*E* + 01 ± 3.77*E* − 01−	1.23*E* + 01 ± 2.53*E* − 01
*F*_15_	3.36*E* + 02 ± 1.41*E* + 02+	3.52*E* + 02 ± 1.08*E* + 02+	4.00*E* + 02 ± 1.15*E* + 02
*F*_16_	8.89*E* + 01 ± 9.84*E* + 01−	1.64*E* + 02 ± 1.26*E* + 02−	5.48*E* + 01 ± 2.92*E* + 01
*F*_17_	1.47*E* + 02 ± 1.56*E* + 02−	1.36*E* + 02 ± 1.12*E* + 02−	6.41*E* + 01 ± 7.58*E* + 01
*F*_18_	9.04*E* + 02 ± 1.14*E* + 00≈	9.04*E* + 02 ± 7.46 − 01≈	9.03*E* + 02 ± 5.59*E* − 01
*F*_19_	9.04*E* + 02 ± 8.02*E* − 01≈	9.04*E* + 02 ± 1.11*E* + 00≈	9.03*E* + 02 ± 2.01*E* − 01
*F*_20_	9.04*E* + 02 ± 2.20*E* − 01≈	9.04*E* + 02 ± 6.97*E* − 01≈	9.03*E* + 02 ± 2.40*E* − 01
*F*_21_	5.00*E* + 02 ± 6.76*E* − 14≈	5.00*E* + 02 ± 7.95*E* − 14≈	5.00*E* + 02 ± 6.76*E* − 14
*F*_22_	8.73*E* + 02 ± 2.19*E* + 01−	8.68*E* + 02 ± 2.16 + 01≈	8.69*E* + 02 ± 1.92*E* + 01
*F*_23_	5.50*E* + 02 ± 8.05*E* + 01−	5.49*E* + 02 ± 7.67*E* + 01−	5.34*E* + 02 ± 3.40*E*−13
*F*_24_	8.66*E* + 02 ± 2.51*E* + 02−	2.00*E* + 02 ± 2.90 − 14≈	2.00*E* + 02 ± 2.90*E* − 14
*F*_25_	2.09*E* + 02 ± 5.22*E* − 01≈	2.10*E* + 02 ± 8.77*E* − 01≈	2.09*E* + 02 ± 2.84*E* − 01
−/+/≈	**10/4/11**	**13/4/8**	

## Data Availability

The data used to support the findings of this study are available from the corresponding author upon request.
